# Supplementary material for: Impact of DC Electric Field Direction on Sedimentation Behavior of Colloidal Particles in Water
Source: Materials (Basel). 2025 Mar 18;18(6):1335. doi: 10.3390/ma18061335 (PMC11944156; doi:10.3390/ma18061335)
Supplement: Supplementary file 1 [file materials-18-01335-s001.zip › materials-3532428-supplementary.pdf]

materials

MDPI

Supplementary Materials

Impact of DC Electric Field Direction on Sedimentation Behavior of Colloidal Particles in Water

Hiroshi Kimura

Department of Chemistry and Biomolecular Science, Faculty of Engineering, Gifu University, Gifu 501-1193, Japan

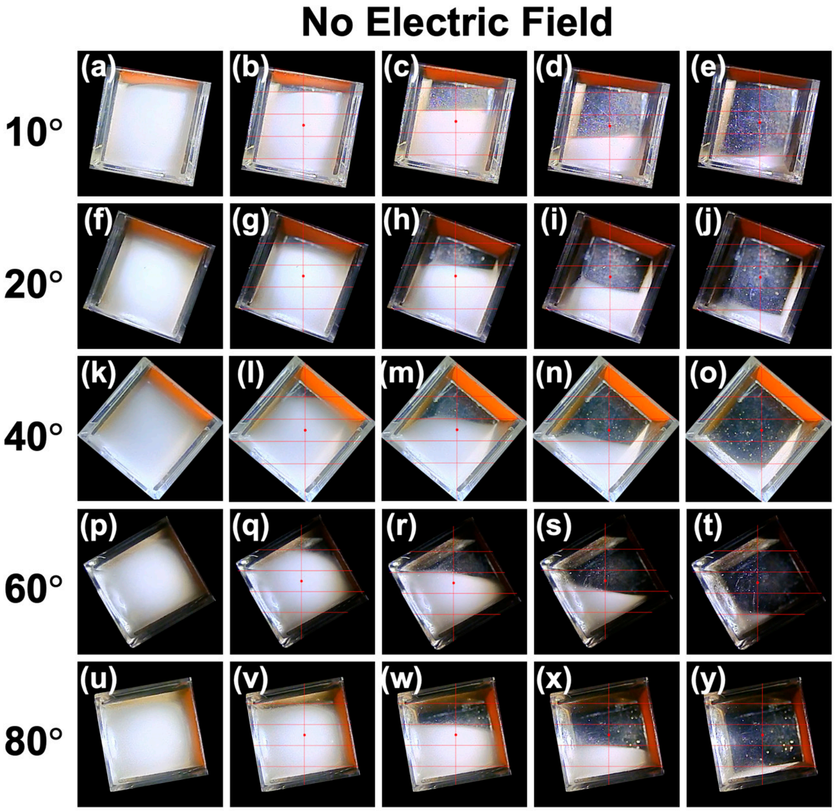

Figure S1. Changes in the dispersion state of PMMA particles at 25°C under no electric field. (**a**–**e**) Inclination angle $\theta = 10°$, (**f**–**j**) $\theta = 20°$, (**k**–**o**) $\theta = 40°$, (**p**–**t**) $\theta = 60°$, (**u**–**y**) $\theta = 80°$. (**a**) Elapsed time $t = 0$, (**b**) 220 s, (**c**) 1040 s, (**d**) 1880 s, (**e**) 2790 s, (**f**) 0, (**g**) 210 s, (**h**) 900 s, (**i**) 1660 s, (**j**) 2470 s, (**k**) 0, (**l**) 260 s, (**m**) 840 s, (**n**) 1570 s, (**o**) 2550 s, (**p**) 0, (**q**) 190 s, (**r**) 740 s, (**s**) 1510 s, (**t**) 2470 s, (**u**) 0, (**v**) 210 s, (**w**) 900 s, (**x**) 1710 s, (**y**) 2650 s.

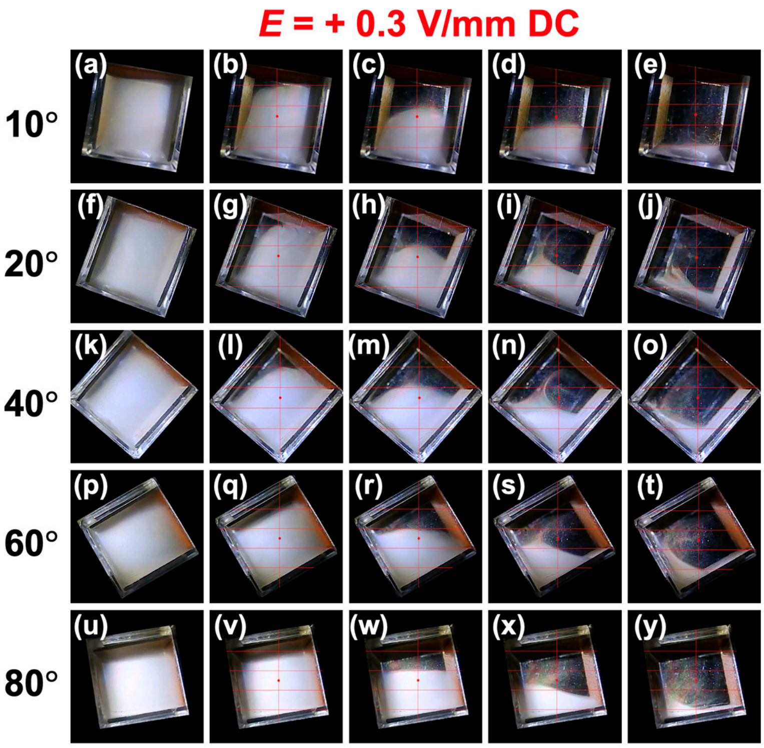

Figure S2. Changes in the dispersion state of PMMA particles at 25°C under E = + 0.3 V/mm DC. (**a**–**e**) Inclination angle θ = 10°, (**f**–**j**) θ = 20°, (**k**–**o**) θ = 40°, (**p**–**t**) θ = 60°, (**u**–**y**) θ = 80°. (**a**) Elapsed time t = 0, (**b**) 100 s, (**c**) 380 s, (**d**) 680 s, (**e**) 1220 s, (**f**) 0, (**g**) 80 s, (**h**) 350 s, (**i**) 690 s, (**j**) 1150 s, (**k**) 0, (**l**) 130 s, (**m**) 360 s, (**n**) 670 s, (**o**) 1080 s, (**p**) 0, (**q**) 40 s, (**r**) 190 s, (**s**) 470 s, (**t**) 810 s, (**u**) 0, (**v**) 10 s, (**w**) 140 s, (**x**) 390 s, (**y**) 760 s.

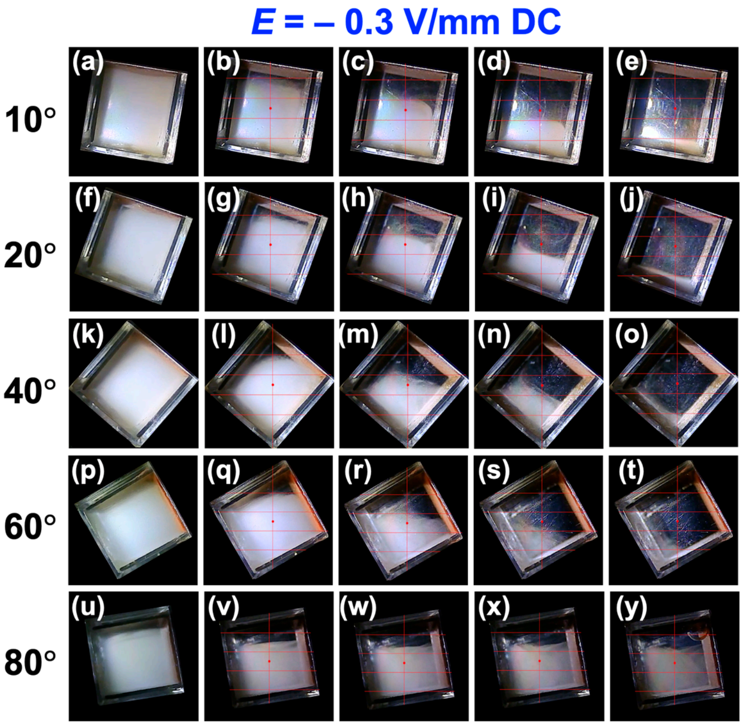

Figure S3. Changes in the dispersion state of PMMA particles at 25°C under E = − 0.3 V/mm DC. (**a**–**e**) Inclination angle θ = 10°, (**f**–**j**) θ = 20°, (**k**–**o**) θ = 40°, (**p**–**t**) θ = 60°, (**u**–**y**) θ = 80°. (**a**) Elapsed time t = 0, (**b**) 360 s, (**c**) 650 s, (**d**) 960 s, (**e**) 1530 s, (**f**) 0, (**g**) 260 s, (**h**) 620 s, (**i**) 960 s, (**j**) 1680 s, (**k**) 0, (**l**) 460 s, (**m**) 2470 s, (**n**) 3650 s, (**o**) 4810 s, (**p**) 0, (**q**) 480 s, (**r**) 2910 s, (**s**) 5980 s, (**t**) 8150 s, (**u**) 0, (**v**) 2000 s, (**w**) 4000 s, (**x**) 6000 s, (**y**) 9650 s.
